# Comparison of Extended-Spectrum *β*-Lactamase-Producing* Escherichia coli* Isolates from Drinking Well Water and Pit Latrine Wastewater in a Rural Area of China

**DOI:** 10.1155/2016/4343564

**Published:** 2016-11-14

**Authors:** Hongna Zhang, Yanxia Gao, Weishan Chang

**Affiliations:** ^1^College of Animal Science and Technology, Shandong Agricultural University, Tai'an 271000, China; ^2^College of Life Sciences, Taishan Medical University, Tai'an 271000, China

## Abstract

The present study was conducted to gain insights into the occurrence and characteristics of extended-spectrum beta-lactamase- (ESBL-) producing* Escherichia coli* (*E. coli*) from drinking well water in the rural area of Laiwu, China, and to explore the role of the nearby pit latrine as a contamination source. ESBL-producing* E. coli* from wells were compared with isolates from pit latrines in the vicinity. The results showed that ESBL-producing* E. coli* isolates, with the same antibiotic resistance profiles, ESBL genes, phylogenetic group, plasmid replicon types, and enterobacterial repetitive intergenic consensus-polymerase chain reaction (ERIC-PCR) fingerprints, were isolated from well water and the nearby pit latrine in the same courtyard. Therefore, ESBL-producing* E. coli* in the pit latrine may be a likely contributor to the presence of ESBL-producing* E. coli* in rural well water.

## 1. Introduction

The use of cephalosporins in clinical practices of humans and animals has contributed to the occurrence of extended-spectrum *β*-lactamase- (ESBL-) producing bacteria across the world [1, 2]. These bacteria are resistant to most beta-lactam antibiotics, such as first-, second-, third-, and fourth-generation cephalosporins, which would increase medical costs and limit treatment options [3, 4].

Many species of Gram-negative bacteria can produce ESBLs, but ESBLs are mainly detected in Enterobacteriaceae, particularly in* Escherichia coli* (*E. coli*) and* Klebsiella* spp. [5]. It is worrisome that not only are ESBL-producing* E. coli* associated with community-acquired infections, especially urinary tract infection [6, 7], but also they have been isolated from food-producing animals and healthy human populations [8, 9].

ESBL-producing* E. coli* in the intestinal tracts of humans and animals are easily excreted into the environment, particularly into surface water bodies. Numerous studies have shown that ESBL-producing* E. coli* can be detected in water bodies [10–13].

Human exposure to ESBL-producing* E. coli* in water bodies may easily occur, for example, when contaminated surface water is used for recreation, for irrigation of crops, or as a drinking water source [14, 15]. Similarly, animal exposure to ESBL-producing* E. coli* in water bodies may also easily occur, for instance, when they drink contaminated surface water [1]. To limit the spread of ESBL-producing* E. coli* via water bodies, it is pivotal to understand the possible contamination sources.

To date, numerous researches about contamination sources of ESBL-producing* E. coli *have been focusing on hospital environments, animal farms, rivers and lakes, and wastewater treatment plants [10, 12, 16–19]. But data about the prevalence and possible contamination sources of ESBL-producing* E. coli* in rural water well in undeveloped regions is very limited.

In undeveloped areas of China, plenty of domestic wells are used to supply drinking water. Impurities from the surface easily enter wells, so the rural wells are relatively easy to be contaminated by bacteria. More importantly, most bacteria, especially ESBL-producing* E. coli*, could contaminate well water coming from fecal material from humans and animals, for instance, from on-site sanitation systems, such as pit latrines and septic tanks [20]. But little information about ESBL-producing* E. coli* contamination in well waters is available in China. This study was therefore conducted to gain insights into the prevalence and characterization of ESBL-producing* E. coli* from drinking well water in the rural area of Laiwu, China, and to explore the role of the nearby pit latrine as a contamination source.

## 2. Materials and Methods

### 2.1. Well Selection and Homeowner Enrollment

The sampling site was located in the rural area of Laiwu city of China, in which the majority of households have both a shallow drinking well (approximately 12–18 m in depth) and a pit latrine in their courtyards, and the distance between them is about 8–10 m. To minimize the influence of surrounding surface water and animal fecal material on the groundwater, water wells located near pit latrines and not within 100 m of rivers/lakes or animal farms were favored for selection in this study. In addition, the distance between two sampled wells is no less than 100 m.

Owners of water wells were sent a letter describing the study and were telephoned two days later to request their participation and confirm that their water well was near a pit latrine.

### 2.2. Sample Collection

Between July and August 2014, a 500 mL well water sample was collected using a sterile bottle from each well (100 wells). During the same period, wastewater samples from 100 pit latrines located near the wells were obtained using the same method. All samples were immediately transported to our lab in an icebox and processed in 6 h.

### 2.3. Isolation and Identification of ESBL-Producing* E. coli*

Each water sample was filtered using a membrane filter (0.45 *μ*m). The filter was then spread on MacConkey agar plate containing cefotaxime (4 *μ*g/mL) and incubated at 37°C overnight. A suspected* E. coli* colony was identified using API 20E (BioMérieux, Marcy-l'Etoile, France).

The suspected ESBL-producing* E. coli* isolates were confirmed by phenotypic confirmatory tests using cefotaxime (30 *μ*g), cefotaxime + clavulanic acid (30 *μ*g/10 *μ*g), ceftazidime (30 *μ*g), and ceftazidime + clavulanic acid (30 *μ*g/10 *μ*g) [21].

### 2.4. Antimicrobial Susceptibility Testing

ESBL-producing* E. coli* isolates were tested for susceptibility to a panel of 16 antibiotics: ampicillin (10 *μ*g), piperacillin (100 *μ*g), amoxicillin/clavulanic acid (20/10 *μ*g), cephalothin (30 *μ*g), cefuroxime (30 *μ*g), ceftazidime (30 *μ*g), ceftriaxone (30 *μ*g), cefepime (30 *μ*g), imipenem (10 *μ*g), meropenem (10 *μ*g), amikacin (30 *μ*g), gentamicin (10 *μ*g), nalidixic acid (30 *μ*g), ciprofloxacin (5 *μ*g), tetracycline (30 *μ*g), and chloramphenicol (30 *μ*g). Antimicrobial susceptibility test was performed according to the CLSI guidelines [21].* E. coli* ATCC 25922 was used as a quality control strain. Isolates from the same sampling site were considered as duplicate strains if they showed the same antibiotic resistance profiles [22].

### 2.5. Detection of Beta-Lactamase Gene

Based on the previously published reference [23], ESBL-producing* E. coli* isolates were subjected to multiplex polymerase chain reaction (PCR) to determine the presence/absence of genes encoding CTX-M, SHV, and TEM. PCR products were sequenced and compared with beta-lactamase gene sequences in the GenBank database and in the Lahey website (http://www.lahey.org/studies).

### 2.6. Phylogenetic Groups

ESBL-producing* E. coli* isolates were assigned to eight groups, A, B1, B2, C, D, E, F, and clade I, according to the published work by Clermont and his colleagues [24].

### 2.7. Plasmid Replicon Typing

According to the previously published references [25, 26], ESBL-producing* E. coli *were subjected to PCR-based plasmid replicon typing. Briefly, PCR amplification was carried out with 18 pairs of primers to recognize FIA, FIB, FIC, HI1, HI2, I1-I*γ*, L/M, N, P, W, T, A/C, K, B/O, X, Y, F, and FIIA in 5 multiplex and 3 simplex reactions.

### 2.8. ERIC-PCR

ESBL-producing* E. coli* isolates were subjected to enterobacterial repetitive intergenic consensus- (ERIC-) PCR [27–29]. ERIC-PCR fingerprints were analyzed using the NTSYSpc software (version 2.02K, Applied Biostatistics, Inc., NY, USA). The dendrogram was constructed based on the average relatedness of the matrix using the algorithm of the unweighted pair-group method (UPGMA) in the SAHN program of the NTSYSpc software.

## 3. Results

### 3.1. ESBL-Producing* E. coli* Isolates

One hundred households with private wells (W1–W100) and pit latrines (P1–P100) in their courtyard were selected to be sampled. A total of 200 samples from 100 wells and 100 pit latrines were obtained in this study. ESBL-producing* E. coli* isolates from the same sampling site were considered as duplicate strains if they showed the same antibiotic resistance profiles [22], and 63 nonduplicate ESBL-producing* E. coli *isolates were obtained in this study, including 23 isolates from 5 wells (W2, W16, W21, W24, and W35; 5/100, 5.0%) and 40 strains from 8 pit latrines (P2, P16, P21, P24, P35, P40, P46, and P56; 8/100, 8.0%) (Table 1).

### 3.2. Antibiotic Resistance Profiles of ESBL-Producing* E. coli*

Similar resistance characteristics of ESBL-producing* E. coli* between two origins were found in this study. All ESBL-producing* E. coli* from well water and pit latrine wastewater were resistant to ampicillin (100%) and cephalothin (100%), and most of the ESBL-producing* E. coli *isolates from well waters were resistant to cefuroxime (95.6%), piperacillin (78.3%), tetracycline (78.3%), nalidixic acid (56.5%), and amoxicillin/clavulanic acid (56.5%); resistance to ciprofloxacin (43.8%) and ceftriaxone (39.1%) was also common; resistance to gentamicin (30.4%), chloramphenicol (30.4%), ceftazidime (26.1%), and cefepime (13.0%) was less frequently observed; none of the strains was resistant to amikacin or carbapenem antibiotics imipenem and meropenem (Table 2).

The majority of ESBL-producing* E. coli *isolates from pit latrine wastewater were resistant to cefuroxime (97.5%), ciprofloxacin (72.5%), piperacillin (70.0%), nalidixic acid (67.5%), gentamicin (62.5%), and tetracycline (57.5%); resistance to ceftriaxone (50.0%) and ceftazidime (45.0%) was also common; but resistance to chloramphenicol (37.5%), amoxicillin/clavulanic acid (32.5%), amikacin (10%), and cefepime (7.5%) was less frequently observed; none of the strains was resistant to carbapenem antibiotics imipenem and meropenem (Table 2).

### 3.3. ***β***-Lactamase Genes of ESBL-Producing* E. coli*

Except that *bla*_CTX-M-3_ gene was restricted to pit latrine wastewater isolates, a similar distribution of *β*-lactamases genes among both sources was found in this study. Twenty-two out of 23 well water isolates (95.6%) and 37 of 40 pit latrine wastewater isolates (92.5%) carried *bla*_CTX-M_ genes. Fifteen out of 23 well water isolates (65.2%) and 30 of 40 pit latrine wastewater isolates (75.0%) carried *bla*_TEM-1_ genes, and most of these isolates combined with *bla*_CTX-M_ genes. Among all ESBL-producing* E. coli* carrying *bla*_CTX-M_ genes, the most prevalent ESBL gene was *bla*_CTX-M-15_. No *bla*_SHV_ genes were detected in this study (Table 3).

### 3.4. Phylogenetic Groups of ESBL-Producing* E. coli*

The phylogenetic group distribution of ESBL-producing* E. coli* from well water and pit latrine wastewater was comparable. 39.1% and 26.1% of ESBL-producing* E. coli* from well water belonged to group A and group B1, respectively. Similarly, 32.5% and 22.5% of ESBL-producing* E. coli* from wastewater belonged to group A and group B1. 26.1% and 8.7% of the well water isolates belonged to groups D and B2, respectively. 27.5% and 17.5% of the wastewater isolates belonged to groups D and B2, respectively (Table 4).

### 3.5. Plasmid Replicon Typing of ESBL-Producing* E. coli*

Of 18 studied plasmid replicon types, 8 were not detected. All isolates carried at least one of the tested plasmid replicon types. The dominant plasmid replicons among well water isolates were FIB (60.1%) and replicon IncI1 (43.5%). Similarly, the most prevalent plasmid replicons among wastewater isolates were IncI1 (70.0%) and FIB (50.0%) (Table 5).

### 3.6. ERIC-PCR Analysis

**Figure 1:**
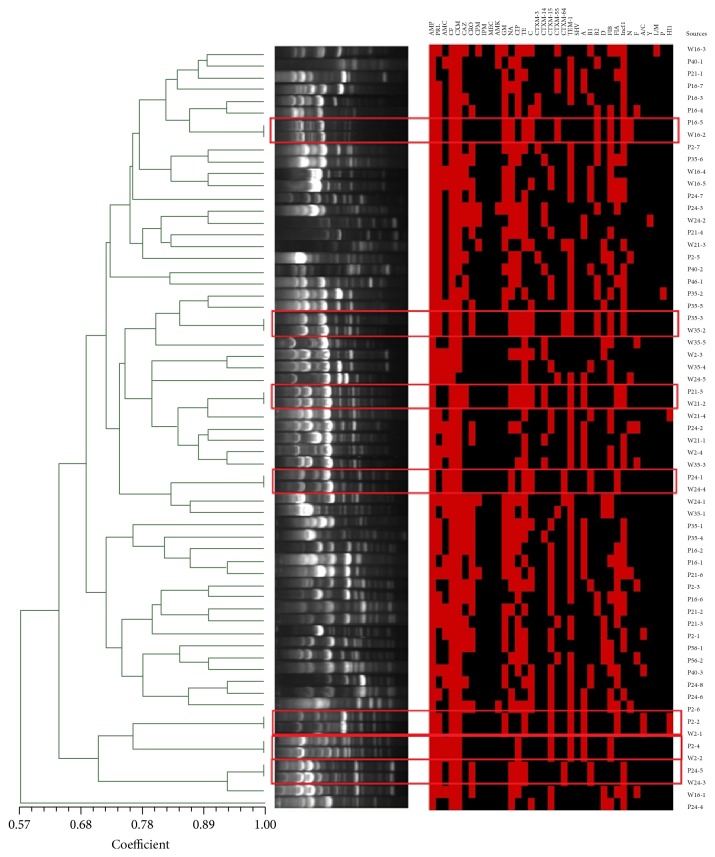
ERIC analysis of ESBL-producing* E. coli *isolates from both well water and pit latrine wastewater. Notes: the dendrogram was constructed based on the average relatedness of the matrix using the algorithm of the unweighted pair-group method (UPGMA) in the SAHN program of the NTSYSpc software. P: pit latrine; W: well water; the red square represents the presence of related phenotypes, genotypes, phylotyping, and plasmid replicons. AMC: amoxicillin/clavulanic acid; CF: cephalothin; CXM: cefuroxime; CAZ: ceftazidime; CRO: ceftriaxone; CPM: cefepime; IPM: imipenem; MEC: meropenem; AMK: amikacin; GM: gentamicin; NA: nalidixic acid; CIP: ciprofloxacin; TE: tetracycline; C: chloramphenicol.

ERIC analysis showed that genetic relatedness of all ESBL-producing* E. coli* isolates ranged between 57.0% and 100.0%. Overall, the isolates from well water and wastewater were extensively intermixed. Of note, seven isolates from five different wells showed 100.0% genetic similarity with isolates from the pit latrine in the same courtyard (Figure 1).

## 4. Discussion 

In this study, there were 7 matches (100.0% similarity) among ESBL-producing* E. coli* from two source groups in the same courtyard. These findings suggested that pit latrine wastewater might be an important source for ESBL-producing* E. coli* isolates from well water.

High resistance rates of ESBL-producing* E. coli* for *β*-lactamases and other antibiotic classes were observed in this study, which is consistent with previous studies about ESBL-producing* E. coli* from water bodies of China or other countries [30–32]. Additionally, relatively high resistance rates to fluoroquinolone (72.5%), ciprofloxacin (62.5%), and gentamicin (62.5%) from wastewater samples were observed, which is related to the fact that those antimicrobials are widely used by humans in China [33, 34]. Of note, compared with pit latrine wastewater, ESBL-producing* E. coli* from well water showed higher rates of resistance against tetracycline (78.3% versus 57.5%), which is frequently used in veterinary practice [33]. Therefore, the higher proportion of tetracycline resistant strains in well water isolates may be associated with the contamination of animal-borne bacteria. Fortunately, only 4.0% of wastewater isolates were resistant to amikacin, and 100.0% of the isolates were susceptible to imipenem and meropenem.

In both source groups, the predominant CTX-M-1 group gene was *bla*_CTX-M-15_, and *bla*_CTX-M-14_ was the most prevalent CTX-M-9 group gene, which is in agreement with several other reports in China [33, 35]. Additionally, *bla*_CTX-M-15_ and *bla*_CTX-M-14_ were used to represent the epidemic CTX-M-1 and CTX-M-9 groups in humans in Asia, respectively [36]. Of note, *bla*_CTX-M-55_ was relatively prevalent in isolates from both sources, which is frequently found in human and food-producing animals in China [34, 37]. *bla*_CTX-M-64_ was detected in isolates from both sources, which could have resulted from recombination of *bla*_CTX-M-55_ and *bla*_CTX-M-14_ [34, 38, 39]. In addition, *bla*_TEM-1-type_ gene was found in both sources, but the lack of identification of TEM-1-type variants by direct sequencing was a limitation of the study.

Several studies have shown that ESBL-producing* E. coli* from patients, poultry, and environmental waters had the same ESBL genes, resistance profiles, and genomic backbone [33, 40]. Similarly, ESBL-producing* E. coli* exhibiting identical antibiotic resistance profiles, ESBL genes, phylogenetic group, plasmid replicon types, and ERIC fingerprints were isolated from well water and the pit latrine in the same courtyard. The result suggested that there is possible transmission of bacteria carrying resistance gene between pit latrine wastewater and well water.

There was a similar phylogroup distribution between both sources, and the prevalent groups were A and B1. Phylogroup A is common in commensal strains from chickens [41]. Also, phylogroup B1 is prevalent among extraintestinal infectious isolates from humans, and group B2 is the most prevalent phylogroup among isolates from humans [39, 42]. The existence of groups B1 and B2 isolates in well water might be an indication of virulent human isolates being spread into the water bodies.

With respect to mobile elements, such as plasmid replicons and *β*-lactamases genes, the high overlap between ESBL-producing* E. coli* from both sources suggested that the transfer of mobile elements may have occurred from fecal wastewater source isolates to well water ones [43].

## 5. Conclusions

Together, these findings showed that ESBL-producing* E. coli* isolates with the same antibiotic resistance profiles, ESBL genes, phylogenetic group, plasmid replicon types, and ERIC-PCR fingerprints were isolated from well water and the nearby pit latrine in the same courtyard. Therefore, it could be concluded that pit latrine wastewater may be an important contributor to the presence of ESBL-producing* E. coli *in well water, and ESBL-producing* E. coli* may disseminate from pit wastewater to well water through long-term permeation or water runoff, especially during heavy rains.

## Figures and Tables

**Table 1:** Sixty-three nonduplicate ESBL-producing *E. coli* isolates from well water and pit latrine wastewater.

Wells	Isolate ID	Number	Pit latrines	Isolate ID	Number
W2	W2-1	4	P2	P2-1	7
	W2-2			P2-2	
	W2-3			P2-3	
	W2-4			P2-4	
				P2-5	
				P2-6	
				P2-7	

W16	W16-1	5	P16	P16-1	7
	W16-2			P16-2	
	W16-3			P16-3	
	W16-4			P16-4	
	W16-5			P16-5	
				P16-6	
				P16-7	

W21	W21-1	4	P21	P21-1	6
	W21-2			P21-2	
	W21-3			P21-3	
	W21-4			P21-4	
				P21-5	
				P21-6	

W24	W24-1	5	P24	P24-1	8
	W24-2			P24-2	
	W24-3			P24-3	
	W24-4			P24-4	
	W24-5			P24-5	
				P24-6	
				P24-7	
				P24-8	

W35	W35-1	5	P35	P35-1	6
	W35-2			P35-2	
	W35-3			P35-3	
	W35-4			P35-4	
	W35-5			P35-5	
				P35-6	
			P40	P40-1	3
				P40-2	
				P40-3	
			P46	P46-1	1
			P56	P56-1	2
				P56-2	

Total		23			40

**Table 2:** Resistance profiles of ESBL-producing* E. coli* from well water and pit latrine wastewater.

Antibiotics	Prevalence of resistance isolates, number (column%)	
	Well water (*n* = 23)	Wastewater (*n* = 40)
Ampicillin (AMP)	23 (100)	40 (100)
Piperacillin (PRL)	18 (78.3)	28 (70.0)
Amoxicillin/clavulanic acid (AMC)	13 (56.5)	11 (32.5)
Cephalothin (CF)	23 (100)	40 (100)
Cefuroxime (CXM)	22 (95.6)	39 (97.5)
Ceftazidime (CAZ)	6 (26.1)	18 (45.0)
Ceftriaxone (CRO)	9 (39.1)	20 (50.0)
Cefepime (CPM)	3 (13.0)	3 (7.5)
Imipenem (IPM)	0 (0)	0 (0)
Meropenem (MEC)	0 (0)	0 (0)
Amikacin (AMK)	0 (0)	4 (10.0)
Gentamicin (GM)	7 (30.4)	25 (62.5)
Nalidixic acid (NA)	13 (56.5)	27 (67.5)
Ciprofloxacin (CIP)	10 (43.8)	29 (72.5)
Tetracycline (TE)	18 (78.3)	23 (57.5)
Chloramphenicol (C)	7 (30.4)	15 (37.5)

**Table 3:** Distribution of *β*-lactamase genes by source group among ESBL-producing *E. coli *isolates from well water and pit latrine wastewater. Note: TEM-1 is not an ESBL.

*β*-Lactamases genes	Prevalence of *β*-lactamases genes, number (column%)	
	Well water (*n* = 23)	Wastewater (*n* = 40)
*bla*_CTX-M-3_	0	4 (10.0%)
*bla*_CTX-M-14_	6 (26.1%)	5 (12.5%)
*bla*_CTX-M-15_	8 (34.8%)	19 (47.5%)
*bla*_CTX-M-55_	3 (13.0%)	6 (15.0%)
*bla*_CTX-M-64_	5 (21.7%)	3 (7.5%)
*bla*_TEM-1-type_	15 (65.2%)	30 (75.0%)
*bla*_SHV-1-type_	0	0

**Table 4:** Distribution of phylogenetic groups among ESBL-producing *E. coli* isolates from well water and pit latrine wastewater.

Phylogenetic group	Prevalence of phylogenetic group, number (column%)	
	Well water (*n* = 23)	Wastewater (*n* = 40)
A	9 (39.1)	13 (32.5)
B1	6 (26.1)	9 (22.5)
B2	2 (8.7)	11 (27.5)
D	6 (26.1)	7 (17.5)

**Table 5:** Distribution of plasmid replicons of ESBL-producing *E. coli *isolates from well water and pit latrine wastewater.

Plasmid replicon	Prevalence of replicon within source group, number (column%)	
	Well water (*n* = 23)	Human fecal waste (*n* = 40)
FIB	14 (60.1)	20 (50.0)
FIA	5 (21.7)	19 (47.5)
IncI1	10 (43.5)	28 (70.0)
N	3 (13.0)	4 (10.0)
F	4 (17.4)	3 (7.5)
A/C	1 (4.3)	3 (7.5)
Y	1 (4.3)	0 (0)
L/M	0	1 (2.5)
P	0	1 (2.5)
HI1	2 (8.7)	1 (2.5)
